# Psychopathological Analysis of Adolescent Girls With Autoimmune Thyroiditis

**DOI:** 10.7759/cureus.50418

**Published:** 2023-12-12

**Authors:** Engin Aydin, Dilek Bingöl Aydin, Gresa Çarkaxhiu Bulut, Şükriye Pınar İşgüven

**Affiliations:** 1 Pediatrics, Health Sciences University Zeynep Kamil Women and Children Diseases Training and Research Hospital, İstanbul, TUR; 2 Pediatric Endocrinology, Cerrahpasa University, İstanbul, TUR; 3 Child and Adolescent Psychiatry, Galata University, İstanbul, TUR; 4 Pediatric Endocrinology, Sakarya University (Retired), İstanbul, TUR

**Keywords:** k-sads, cdi, thyroiditis, depression, autoimmunity, anxiety, hashimoto disease, female, adolescent, child

## Abstract

Aim: Clinical studies indicate that there is an association between high levels of thyroid autoantibodies and psychiatric disorders, independent of impairment of thyroid function. Therefore, we aimed to investigate the association between thyroid autoimmunity and mood disorders in euthyroid girls with Hashimoto’s thyroiditis (HT) in a case-control study.

Material and methods: We recruited 82 participants: 41 pubertal female patients with thyroiditis from endocrine outpatient clinics and a control group of 41 healthy pubertal girls from the University Hospital. Age ranged from 12 to 18 years; the diagnosis of HT was based on high levels of anti-TPO and/or anti-Tg antibodies associated with a hypoechogenic or normal thyroid ultrasound pattern. Other comorbidities known to affect mental and physical health were exclusion factors. All participants underwent a complete thyroid evaluation, assays of serum-free T4, TSH, anti-TPO antibodies, anti-Tg antibodies, and thyroid ultrasonography. They were then referred to a child psychiatrist. A psychiatric diagnosis was made in two steps. First, the Children’s Depression Inventory (CDI) and Screen for Child Anxiety-Related Emotional Disorders (SCARED) tests were implemented according to the DSM-IV diagnostic criteria to be calculated. Second, the same psychiatrist conducted a K-SADS-PL semi-structured interview while unaware of the children’s data.

Results: There was no significant difference in CDI score between patients with and without HT (*p* = 0.47). Patients with HT had significantly higher SCARED scores than patients without HT (*p* < 0.05). In the SCARED test, the subcategories of separation anxiety and social anxiety were significantly higher in the HT group (*p* = 0.04 and *p* = 0.01, respectively). During the K-SADS interview by the attending child psychiatrist, psychopathology diagnoses were detected in 27 of 41 patients (66%) with HT and in 8 of 41 individuals (19.5%) in the control group. Psychopathology was significantly higher in the HT group (*p* < 0.01). The incidences of depressive disorder, generalized anxiety disorder, and social phobia were significantly higher in the HT group than in the control group (*p* < 0.05). Multivariate logistic regression analysis revealed that the anti-TPO value was the most significant independent risk factor for the presence of depressive disorder (*p* < 0.01).

Conclusion: This study described severe psychometric impairment in patients with euthyroid HT. We have demonstrated that autoimmune thyroid diseases, even in a euthyroid state, are associated with psychiatric disorders.

## Introduction

Dr. Hakaru Hashimoto originally identified a persistent thyroid condition in 1912. Autoimmune thyroiditis (AIT) affects 0.3-1.5/1000 persons per year, with women being more frequently affected (4-10 times more frequently) than males [1]. Hashimoto thyroiditis (HT) is the most common type of autoimmune thyroiditis. The majority of the diagnosis, ultrasonographic parenchyma that is hypoechogenic and dyshomogeneous positive autoantibodies, indicates a thyroid condition [2]. The development of AIT is influenced by both B and T cell activation. Hence, it can be inferred that one of the most important factors in the development of thyroid autoimmunity is the interaction between cellular immunity and humoral immunity [3]. Affective disorders are more prevalent in patients with autoimmune diseases [2,4-6]. Psychiatric diseases and autoimmune reactions appear to have a common underlying aberration in the immuno-endocrine system, which is not easily correctable [6]. Depression and anxiety are quite frequent among young people, especially adolescents. One in six hospitalized youngsters in the United States is diagnosed with major depression [7]. Causes, diagnosis, and treatment of teenage psychopathology are subjects of study in several scientific disciplines, including neuroendocrinology [5]. Depressive and psychopathological changes do not only arise from adolescence but also from autoimmune diseases [8-10]. Evidence suggests that abnormalities in the hypothalamic-pituitary-adrenal (HPA) axis have a significant role in the pathophysiology of major depressive disorder in adults. Considering the pediatric population, it is important to emphasize that HPA-axis activity varies with developmental phase, with stronger activity in childhood and larger chemical reactions in adolescence [11,12]. While studies in the literature examine the relationship between autoimmunity in patients in the hypothyroidism or subclinical hypothyroidism phase, we aimed to examine the relationship between autoimmunity and psychiatric symptoms in euthyroid patients who do not use medication to eliminate the negative effects of low hormone levels [13,14]. This study aimed to determine the relationship between psychosomatic symptoms, examined through semi-structured interviews and psychiatric evaluation, and autoimmunity in adolescent girls with euthyroid Hashimoto thyroiditis.

## Materials and methods

Participants' inclusion criteria for the study

Participants included 41 pubertal female patients with HT. Adolescent girls between the ages of 12 and 18 (18 years old are not included) who applied to the pediatric endocrinology outpatient clinic participated in the study. These individuals had normal levels of thyroid-stimulating hormone (TSH) and free T4, tested positive for thyroid autoantibodies (anti-TPO and anti-Tg), had thyroiditis (heterogeneous and coarse echotexture; hypoechogenicity with or without coarse septations from fibrous bands) detected by ultrasound, and were not undergoing any therapy. Thyroiditis ultrasonography findings accompanied by one or two antibody positivities have been accepted for the diagnosis of Hashimoto's disease in adolescents. The control group included 41 healthy pubertal adolescent girls who visited the Pediatric and Pediatric Endocrinology Outpatient Clinic, matched the age and body mass index (BMI) of the HT group, had normal thyroid function and negative autoantibodies, and had thyroid ultrasonography that was evaluated as normal. All participants were age-adjusted, and their height and weight were measured. Weight measurements were made with a calibrated scale sensitive to 100 g, and height measurements were made with a calibrated stadiometer sensitive to 1 mm (SECA, model 220, Hamburg, Germany). BMI and standard deviation scores (SDS) were calculated by https://www.ceddcozum.com from the Turkish Pediatric Endocrinology and Diabetes Society© Neyzi reference [15]. Individuals with endocrine, metabolic, neurological, or chronic diseases other than HT; those with active psychiatric disease at the time of the study; those using medication; and those with a physical disability were not included in the study.

Laboratory study method

Blood samples were obtained to assess the blood glucose, insulin, free thyroxine (fT4), TSH, thyroid autoantibodies (anti-TPO and anti-Tg), cortisol, and homeostatic model assessment-insulin resistance (HOMA-IR) values of all HT and control patients. We collected samples venously after 12 hours of fasting, around 8 a.m. We used Beckman Coulter AU 5800 and Siemens BNII autoanalyzers in the biochemistry and microbiology laboratory at University Hospital for the analyses. The thyroid hormones were measured using the Abbott® Architect Free T3 Reagent Assay (USA), the Abbott® Architect Free T4 Reagent Assay (USA), and the Abbott® Architect TSH Assay (USA). The serum samples were evaluated using the Chemiflex technique on the Abbott® Architect i2000 autoanalyzer located in the biochemistry laboratory of the hospital.

Application of psychological surveys and examination

After the thyroid evaluation, relevant study participants were referred to an outpatient clinic and evaluated by the same child and adolescent psychiatrist. Two scales were assessed, and a semi-conducted face-to-face interview was conducted. First, the Children’s Depression Inventory (CDI) survey was applied to the participants. The CDI is a self-reported scale that was developed by Kovacs to determine the severity of depression in children and adolescents [16]. The scale was adapted into Turkish by Öy [17]. CDI is a good measurement tool that determines the severity of depression. Each item in the test evaluates the last two weeks. The highest score on the scale is 54. No special training is required to apply the scale.

The second test we administered was the Screen for Child Anxiety-Related Emotional Disorders (SCARED), developed by Birmaher et al. [18]. SCARED, which was validated and found to be reliable in Turkish by Çakmakçı [19], has a parent form and a child form. A score of 25 or above on SCARED, which consists of 41 items in total, is considered a warning for anxiety disorder. The scale includes somatic/panic, generalized anxiety, separation anxiety, social anxiety, and school fear subscales.

Last, the Kiddie-SADS-Lifetime Version (K-SADS-PL) was implemented by a doctor. This evaluation consists of three parts. The first part is an unstructured initial interview. The second component is a diagnostic screening interview that assesses approximately 200 symptoms. If there are positive symptoms present in this section, further scoring is conducted to validate the diagnosis in the fields of affective disorders, psychotic disorders, anxiety disorders, behavioral disorders, drug abuse disorders, and other disorders [20-22]. This tool was developed by Kauffman et al. A validity and reliability study of the Turkish version of K-SADS-PL was conducted by Gökler et al. [23].

Data analysis

Statistical analyses were conducted using SPSS® 23.0 (IBM Corp., Armonk, NY). For continuous variables, descriptive statistics were provided as mean ± standard deviation and median with an interquartile range (IQR) ranging from 25% to 75%, contingent on the distribution of the variables. We used histograms and the Kolmogorov-Smirnov test to assess the normality of variables. The mean, standard deviation, and median were utilized for descriptive analyses. To compare scaled data in the HT and control groups, an independent sample t-test was employed for normally distributed variables, and a Mann-Whitney U test was employed for non-normally distributed variables. Student’s t-tests were used to assess normally distributed values. We employed Fisher’s exact test or Pearson’s chi-square test to compare categorical variables by group. Logistic regression was used to determine variables positively correlated with anti-TPO in the HT group and control group. Multivariate analysis included statistically significant and clinically important univariate variables. In all statistical comparisons, p-values less than 0.05 were considered significant.

Ethical approval and informed consent

Adolescents in the patient and control groups were informed about the study. Before the study, a written informed consent form was obtained from both the child and her parents. Ethical approval was received from the ethics committee of Sakarya University Faculty of Medicine (No. E.4621:71522473/050.01.04/76). Appointment times for participant history, physical examination, blood collection, and psychological evaluation were arranged before the psychiatry interview.

## Results

A total of 82 subjects, with a mean age of 15.5 ± 1.7 years, participated in the study. Table 1 shows the age, height, weight, BMI, and calculated SDS of all study participants. The mean BMI of the participants was 21.01 ± 3.1 kg/m^2^.

**Table 1:** Age and anthropometric measurements of all participants ^†^Mean ± standard deviation, ^‡^median [IQR: Q1–Q3], *independent samples t-test, **Mann-Whitney U test. BMI: body mass index, SDS: standard deviation score.

Variables	Overall (n = 82)	Control group (n = 41)	Study group (n = 41)	p-value
Age (years)^†^	15.5 ± 1.7	15.8 ± 1.6	15.2 ± 1.7	0.120*
Weight (kg)^†^	54.3 ± 9.6	52.8 ± 8.2	56.2 ± 7.2	0.168*
Height (cm)^†^	160.5 ± 7.4	160.3 ± 8.9	160.3 ± 7.2	0.997*
BMI (kg/m^2^)^†^	21.01 ± 3.1	20.4 ± 2.4	21.7 ± 3.6	0.090*
BMI SDS^‡^	−0.2 [−1 to 0.7]	−0.5 [−1.2 to 0.3]	0.3 [−0.8 to 0.9]	0.069**
Weight SDS^‡^	−0.2 [−1.1 to 0.5]	−0.4 [−1.2 to 0.3]	0.1 [−0.9 to 0.8]	0.103**
Height SDS^‡^	0.1 [−0.9 to 0.8]	0.0 [−0.8 to 0.8]	0.1 [−0.8 to 0.8]	0.846**

There were no significant differences in age or anthropometric values between the study and control groups. The groups were similar in terms of age, weight, height, BMI, and the SDS of weight, height, and BMI (p > 0.05 for all).

Laboratory results are given in Table 2. The values of glucose, HOMA-IR, insulin, cortisol, and free T4 were similar in both groups (p > 0.05 for all). However, the median TSH in the study group was significantly higher than that in the control group (p < 0.001). TSH measurements in both groups were within the normal reference laboratory range.

**Table 2:** Laboratory features of the study groups ^†^Mean ± standard deviation, ^‡^median [IQR: Q1–Q3], ¥: n (%), *independent samples t-test, **Mann-Whitney U test. HOMA-IR: homeostatic model assessment-insulin resistance, TSH: thyroid-stimulating hormone, TPO: thyroid peroxidase, Tg: thyroglobulin. *Reference ranges*: fT3 (2.62–5.69 pmol/L), fT4 (9.00–19.04 pmol/L), TSH (0.35–4.94 µIU/mL), anti-TPO (0–5, based on 61 IU/mL), anti-Tg (0–4.11 IU/mL), and cortisol (3.7–19.4 µg/dL).

Variables	Overall (n = 82)	Control group (n = 41)	Study group (n = 41)	p-value
Glucose (mg/dL)^†^	88.6 ± 9.3	87.2 ± 10.5	89.9 ± 7.8	0.201*
HOMA-IR^‡^	2.0 [1.4–2.6]	1.8 [1.3–2.6]	2.1 [1.6–2.6]	0.438**
Insulin (µu/mL)^†^	9.5 ± 4.2	9.3 ± 5.0	9.6 ± 3.5	0.732*
Cortisol (µg/dL)^†^	9.0 ± 3.7	8.0 ± 2.8	9.7 ± 4.2	0.099*
TSH (mIU/mL)^‡^	1.7 [1.0–2.3]	1.3 [1.0–1.6]	2.1 [1.7–3.7]	<0.001**
Free T4 (nmol/mL)^†^	13.1 ± 1.6	13.1 ± 1.3	13.2 ± 1.8	0.725*
Anti-TPO (IU/mL)^‡^	1.7 [0.4–211.5]	0.4 [0.2–0.6]	220.7 [65.7–787.3]	<0.001**
Anti-Tg (IU/mL)^‡^	7.1 [1.6–44.7]	1.7 [1.3–3.1]	46.7 [15.6–119.4]	<0.001**

The survey results for the two groups are given in Table 3. The median CDI scores in the control and study groups were similar (p = 0.546). The mean SCARED score was 35.1 ± 13.4 in the study group, and it was statistically significantly higher than that of the control group (p = 0.027). More than half of the patients (54.9%) reported panic attacks as the most common psychiatric symptom on self-reported scales. There was a significant difference in the presence of social anxiety between the groups (p = 0.024). Social anxiety was observed in 53.7% of the patients in the study group, whereas 26.8% of the control patients reported social anxiety. Although panic attacks and separation anxiety were more common in the study group, there were no significant differences between the groups (p = 0.375 and p = 0.072, respectively).

**Table 3:** Clinical features of the study and control groups ^†^Mean ± standard deviation, ^‡^median [IQR: Q1–Q3], ^¥^n (%), *independent samples t-test, **Mann-Whitney U test, ***Pearson chi-square♣/Fisher’s exact test♠. CDI: Children’s Depression Inventory, SCARED: Screen for Child Anxiety-Related Emotional Disorders.

Variables	Overall (n = 82)	Control group (n = 41)	Study group (n = 41)	p-value
CDI score^‡^	14.5 [9.0–19.0]	14.0 [8.0–19.0]	15.0 [10.0–20.0]	0.546**
SCARED score^†^	32.1 ± 12.3	29.2 ± 10.3	35.1 ± 13.4	0.027*
Psychiatric symptoms^¥^				
Panic attacks	45 (54.9)	20 (48.8)	25 (61.0)	0.375***
Generalized anxiety	28 (34.1)	14 (34.1)	14 (34.1)	0.999***
Separation anxiety	33 (40.2)	12 (29.3)	21 (51.2)	0.043***
Social anxiety	33 (40.2)	11 (26.8)	22 (53.7)	0.024***
School anxiety	13 (15.9)	7 (17.1)	6 (14.6)	0.999***
Psychiatric diagnosis^¥^				
Panic disorder	3 (3.7)	0 (0.0)	3 (7.3)	0.241***^♠^
Attention deficit hyperactivity dis.	10 (12.2)	2 (4.9)	8 (19.5)	0.088***^♣^
Depressive disorder	14 (17.1)	2 (4.9)	12 (29.3)	0.003***^♣^
Generalized anxiety disorder	11 (13.4)	1 (2.4)	10 (24.4)	0.004***^♣^
Social phobia	17 (20.7)	4 (9.8)	13 (31.7)	0.014***^♣^
Separation anxiety	6 (7.3)	1 (2.4)	5 (12.2)	0.201***^♠^
Obsessive-compulsive disorder	2 (2.4)	0 (0.0)	2 (4.9)	0.494***^♠^

There was a significant difference in the presence of depressive disorder (p = 0.003), generalized anxiety disorder (p = 0.004), and social phobia (p = 0.014) between groups, as determined by semi-structured diagnoses by the physician. There were 12 patients (29.3%) with depressive disorder in the study group, compared to two patients (4.9%) in the control group. There were 10 patients (24.4%) with generalized anxiety disorder in the study group, compared to one patient (2.4%) in the control group. There were 13 patients (31.7%) with social phobia in the study group, compared to four patients (9.8%) in the control group. Although more patients were diagnosed in the study group, due to limitations and a small sample size, other diagnosed diseases were not statistically higher in the study group than in the control group.

Additionally, when the patient and control groups with and without symptoms are compared according to the scales in Table 4, it is seen that there is a statistically significant difference (p < 0.001) between the CDI and SCARED scores in all groups between participants with and without symptoms, as expected.

**Table 4:** Comparison of demographics and laboratory findings in participants with and without psychiatric symptoms in the control and study groups ^†^Mean ± standard deviation, ^‡^median [IQR: Q1–Q3], ^¥^n (%), *independent samples t-test, **Mann-Whitney U test. BMI: body mass index; SDS: standard deviation score; CDI: Children’s Depression Inventory; SCARED: Screen for Child Anxiety-Related Emotional Disorders; HOMA-IR: homeostatic model assessment-insulin resistance; TSH: thyroid-stimulating hormone.

Variables	Overall (n = 82)			Control group (n = 41)			Study group (n = 41)		
	Psychiatric symptoms		p-value	Psychiatric symptoms		p-value	Psychiatric symptoms		p-value
	Negative (n = 65)	Positive (n = 17)		Negative (n = 9)	Positive (n = 32)		Negative (n = 33)	Positive (n = 8)	
BMI SDS^‡^	−0.2 [−1.0 to 0.7]	0.3 [−0.5 to 0.7]	0.461**	0.1 [−0.5 to 0.4]	−0.5 [−1.2 to 0.4]	0.413	0.1 [−0.8 to 0.9]	0.6 [−0.7 to 1.1]	0.622**
Weight SDS^‡^	−0.2 [−1.1 to 0.4]	0.1 [−0.5 to 0.6]	0.342**	−0.3 [−0.5 to 0.4]	−0.8 [−1.2 to 0.3]	0.284	−0.1 [−0.8 to 0.7]	0.1 [−0.4 to 1.1]	0.599**
Height SDS^‡^	−0.1 [−0.9 to 0.8]	0.4 [−0.6 to 0.9]	0.206**	0.4 [−0.6 to 0.9]	−0.2 [−0.9 to 0.5]	0.498	−0.1 [−0.9 to 0.8]	0.4 [−0.0 to 0.7]	0.300**
CDI score^‡^	16.0 [12.0–20.0]	5.0 [3.0–12.0]	<0.001**	4.0 [2.0–8.0]	16.0 [11.8–19.2]	<0.001	16.0 [12.0–21.0]	8.0 [4.5–13.0]	0.011**
SCARED score^†^	36.4 ± 9.8	16.1 ± 5.3	<0.001*	19.0 [14.0–21.0]	31.0 [27.8–35.2]	<0.001	40.0 [34.0–48.0]	15.0 [12.8–17.0]	<0.001**
Laboratory findings									
Glucose (mg/dL)^†^	88.0 [84.0–97.0]	84.0 [81.0–89.0]	0.027**	83.0 [77.0–86.0]	87.0 [82.8–94.8]	0.115	92.0 [85.0–97.0]	84.5 [83.0–89.8]	0.130**
HOMA-IR^‡^	2.1 [1.4–2.7]	1.6 [1.4–2.1]	0.139**	1.6 [1.4–1.6]	2.0 [1.2–2.9]	0.147	2.1 [1.6–2.7]	1.9 [1.6–2.2]	0.518**
Insulin (µg/mL)^†^	9.7 ± 4.2	8.9 ± 4.5	0.526*	7.2 [6.7–7.5]	9.5 [5.8–12.3]	0.425	9.6 [7.3–11.3]	8.8 [7.3–10.6]	0.718**
Cortisol (µg/dL)^†^	9.0 ± 3.7	9.2 ± 4.2	0.887*	6.9 [4.7–10.0]	7.1 [6.2–9.7]	0.548	8.9 [6.2–11.1]	12.2 [9.4–13.4]	0.411**
TSH (mIU/mL)^‡^	1.7 [1.0–2.4]	1.6 [1.3–2.0]	0.886**	1.4 [1.0–1.6]	1.3 [0.9–1.7]	0.450	2.2 [1.7–3.8]	1.9 [1.7–2.2]	0.469**
Free T4 (nmol/mL)^†^	13.0 [12.2–13.7]	12.7 [12.1–13.7]	0.991**	12.6 [12.0–13.6]	13.2 [12.1–13.8]	0.671	12.7 [12.3–13.7]	13.4 [12.5–13.7]	0.532*

There were no significant correlations between anti-TPO and anti-Tg values and the CDI and SCARED scores in the study group (p > 0.05 for all) (Table 5).

**Table 5:** Correlations of anti-TPO and anti-Tg with CDI and SCARED scores in the study group TPO: thyroid peroxidase; Tg: thyroglobulin; CDI: Children’s Depression Inventory; SCARED: Screen for Child Anxiety-Related Emotional Disorders.

Variable	Variable	Spearman’s rho	p-value
Anti-TPO (IU/mL)	CDI score	0.087	0.590
Anti-TPO (IU/mL)	SCARED score	0.186	0.245
Anti-Tg (IU/mL)	CDI score	0.102	0.527
Anti-Tg (IU/mL)	SCARED score	−0.167	0.297

Multivariate logistic regression analysis revealed that the anti-TPO value was a significant independent risk factor for the presence of depressive disorder (adjusted odds ratio (OR) = 1.002, 95% confidence interval (CI) 1.001-1.004, p < 0.011) (Table 6).

**Table 6:** Logistic regression analysis of the factors predicting the presence of depressive disorder Dependent variable: depressive disorder; OR: odds ratio; CI: confidence interval; CDI: Children’s Depression Inventory; SCARED: Screen for Child Anxiety-Related Emotional Disorders; anti-TPO: thyroid peroxidase antibody; anti-Tg: thyroglobulin antibody. Crude: enter regression method, adjusted: backward Wald regression analysis.

Variable	Crude OR (95% CI)	Crude p-value	Adjusted OR (95% CI)	Adjusted p-value
CDI score	1.202 [1.033–1.399]	0.017	-	-
SCARED score	1.007 [0.923–1.099]	0.872	-	-
Anti-TPO	1.002 [1.00–1.004]	0.047	1.002 [1.001–1.004]	0.011
Anti-Tg	1.004 [0.99–1.008]	0.112	-	-

## Discussion

A patient and control group consisting of the same gender and similar ages was selected to ensure that psychiatric symptoms and thyroiditis diagnosis were not affected by age and gender. Since many mental illnesses and thyroid function issues share symptoms and outcomes, we wanted to conduct a comparative analysis of patients in our study and control groups, who were all euthyroid, that was not dependent on thyroid function [5,6,8,10,14].

Diagnosed depressive disorder, generalized anxiety disorder, and social phobia were more commonly diagnosed in adolescent girls with HT in our study. In a study conducted on adults in Germany, pathologically increased anti-TPO levels were found to be significant among patients with depression and schizophrenia. In a sex- and age-adjusted logistic regression, the OR for AIT was tenfold higher in mono- or bipolar patients with depression compared with patients with schizophrenia [24]. Jørgensen and Feldt-Rasmussen claim that “the clinical manifestations of depression frequently exhibit similarities to those of hypothyroidism and may indicate a state of reduced thyroid hormone activity in the brain, even in the presence of normal thyroid hormone levels throughout the body” [25]. Similarly, even though the patients were in an euthyroid state in our study, the child and adolescent psychiatrist's diagnosis revealed that depressive symptoms were widespread and varied between groups.

It is also important to perform a psychiatric evaluation in the monitoring of thyroiditis auto-inflammation, which is a chronic disease that will be helpful due to chronicity of the disease nature causing associated long-term psychiatric manifestations. Regarding this, our semi-structured doctor interview revealed that depressive disorder had a prevalence of 29.3%, generalized anxiety disorder of 24.4%, and social phobia of 31.7%, and these values were higher in HT patients than in control patients. Our findings are in accordance with previous studies. Müssig et al. reviewed the prevalence of neuropsychiatric diseases and neuropsychological testing of anti-TPO prevalence for symptomatic distress in HT patients; Degner et al. found significantly elevated anti-TPO in patients with schizophrenia (5.3%) and in those with uni- or bipolar depression (32.7%); and Giynas et al. showed that "their HT group exhibited a substantially increased prevalence of depression, obsessional compulsive disorder, and panic disorder compared to their control group” [14,26,27].

In addition, the CDI and SCARED self-reported measures are screening tools for the evaluation of mood and feelings [16,18,28,29]. Their use for diagnosis and treatment is valuable, but psychiatric diagnosis of children and adolescents using semi-structured interviews is more accurate. The SCARED score was statistically significant between our study and control groups than the CDI survey, whereas the median CDI scores in the control and study groups were similar. The mean SCARED score was 35.1 ± 13.4 in the study group, which was statistically higher than that of the control group.

While the connection between hypothyroidism and neurodevelopmental and emotional states has been proven, studies indicate that the functioning of the thyroid axis in the absence of hypothyroidism, its impact on emotions, and the architecture of the limbic system are still not well understood [4,8]. Waat et al. administered the health-related quality of life (QoL) thyroid-specific QoL questionnaire ThyPRO to AIT patients and revealed that, based on a pairwise regression analysis, the thyroid peroxidase antibody (TPOAb) level was associated with some outcomes (depressivity, anxiety, and emotional susceptibility), yet thyroid functioning tests were not [13].

Animal research shows that HT causes neuroinflammation and affective changes in euthyroid mice [30]. We conducted our study to see whether the euthyroid state only affects neuromodulation or not. Our results show only the relationship of anti-TPO with mood and emotional condition through regression analysis of the questionnaire scores obtained in relation to depressive disorder.

This study had some limitations and strengths. First, we conducted the study only on girls (in whom HT is seen 4-10 times more frequently than boys) and compared them with psychopathologically healthy individuals. By including a particular sample of girls, we avoided potentially confounding differences in gender and stage of puberty. Our small sample size is a limitation of our study.

## Conclusions

We suggest that even if there is no subclinical hypothyroidism, the autoimmune thyroid structure causes psychosomatic diseases and symptoms. It is likely that the immunological effects of HT will emerge as more specific and detailed studies are conducted.
